# Dr. Latta’s Circular

**Published:** 1850-01

**Authors:** M. M. Latta

**Affiliations:** Member of Com. for Indiana; Goshen, Ind.


### ARTICLE VII.

### DR. LATTA'S CIRCULAR.

[We call the attention of our readers in Indiana, to the following circular. The subject is one of great importance, and we hope the profession will give the committee all the assistance possible in furtherance of its design. Every physician in Indiana should esteem it his duty to furnish Dr. Latta any facts he may have bearing on this point, and his opinion as to the best mode of remedying the evil.]

To Dr. E. G. Meek:

*Dear Sir :*—At the last meeting of the American Medical Association, a Committee of two from each State and Territory was appointed, whose duty it was to "note all facts that come to their knowledge, with regard to the *Adulteration or Sophistication of Drugs, Medicines,*

*&c*,, and to report them through the Committee at the next meeting."

In pursuance of this Resolution, Dr. Meeker, of Laporte, and myself have been appointed members of the committee for this State. Prof. Huston, of Philadelphia, (who is Chairman of the Committee,) in a letter notifying us of our appointment, says that the subject was deemed of the highest importance by the Association, and expresses a hope that we will investigate the matter as fully as circumstances permit, and communicate the facts to him, and such suggestions as we may think proper to add.

Agreeing with the Association in regard to the importance of the subject, and desiring to perform the duty assigned me, in a manner likely to promote the great objects in view, I have ventured to solicit your co-operation. The various Colleges of Pharmacy have entitled themselves to public regard, by their unwearied efforts to detect and expose the practice of Importing bad Drugs into the country. But the evil has been domesticated. It is now, to a great extent, beyond their reach. Large quantities of Drugs that are worthless, or worse, are undoubtedly manufactured in this country; and since the passage of a law by Congress, prohibiting their introduction from abroad, the temptation to adulterate or sophisticate valuable preparations, has been greatly increased, on this side of the water. Nearly all inert or deleterious compounds, whether of Foreign or Domestic manufacture, are expected to find a Western market, and are actually consumed, by our population. Our opportunities for observation are thus rendered extensive, and if properly made use of, cannot fail in removing one of the greatest causes of mortification to the Physician, and of disaster to the suffering.

I therefore respectfully request you to inform me of any such practices as may have come to your knowledge, particularly in regard to Medicines in most common use, as the various preparations of Mercury, Opium, Iodine, Bark, and the Essential Oils, of Nitrate of Silver, Spirits of Nitre, Strychnia, the Vegetable Extracts, &c., together with your opinion as to the best means of removing the evil altogether.

Any information on these subjects, which you may be kind enough to furnish, shall be immediately forwarded to the Chairman of the Committee, and fairly accredited.

As prompt a reply as it will suit your convenience to make, will greatly oblige

Your Ob't. Servant,

M. M. LATTA,
Member of Com. for Indiana.

Goshen, Ind., Nov. 13, 1849.

